# Critical role and its underlying molecular events of the plasminogen receptor, S100A10 in malignant tumor and non-tumor diseases

**DOI:** 10.7150/jca.36203

**Published:** 2020-01-01

**Authors:** Chunyuan Li, Yi Ma, Fei Fei, Minying Zheng, Zugui Li, Qi Zhao, Jiaxing Du, Kai Liu, Rui Lu, Shiwu Zhang

**Affiliations:** 1 Department of Pathology, Tianjin Union Medical Center, Tianjin, P.R. China.; 2 Department of ophthalmology, Tianjin Union Medical Center, Tianjin, P.R. China; 3 Graduate School, Tianjin University of Traditional Chinese Medicine, Tianjin, P.R. China; 4 Tianjin Medical University, Tianjin, P.R. China

**Keywords:** plasminogen receptor S100A10, Annexin A2, malignant tumor, non-tumor diseases

## Abstract

S100A10 is a small molecular weight protein expressed in the cytoplasm of many cells and one of the members of the S100 protein family that binds calcium and forms the largest subgroup of EF-hand proteins. The regulatory processes of S100A10 are complicated. S100A10 participates in the regulation of a variety of tumor and non-tumor diseases through cascade reactions with multitudinous signaling molecules. In malignant tumors, such as acute promyelocytic leukemia (APL) and lung cancer, S100A10 is likely involved in their progression, including invasion and metastasis through the regulation of plasmin production and subsequent plasmin-dependent stimulation of other proteases, such as matrix metalloproteinase (MMP)-2 and -9. Both the plasmin and MMPs are capable of inducing degradation of the extracellular matrix (ECM) and basement membrane, which is a critical step for tumor progression. In non-tumor diseases, the distribution of S100A10 in the brain and its interaction with 5-hydroxytryptamine 1B (5-HT1B) receptor, an important mediator in the central nervous system that maintains a dynamic balance of the neurotransmitters, correlates with depression-like behavior. S100A10 also participates in inflammatory responses through the regulation of peripheral macrophage migration to the inflammatory sites, which depends on the generation of plasmin and other proteinases at the surface of macrophages. Considerable attention should be paid to understand the significant role of S100A10 in the modulation of malignant tumor and non-tumor diseases.

## Introduction

**Figure 1:**
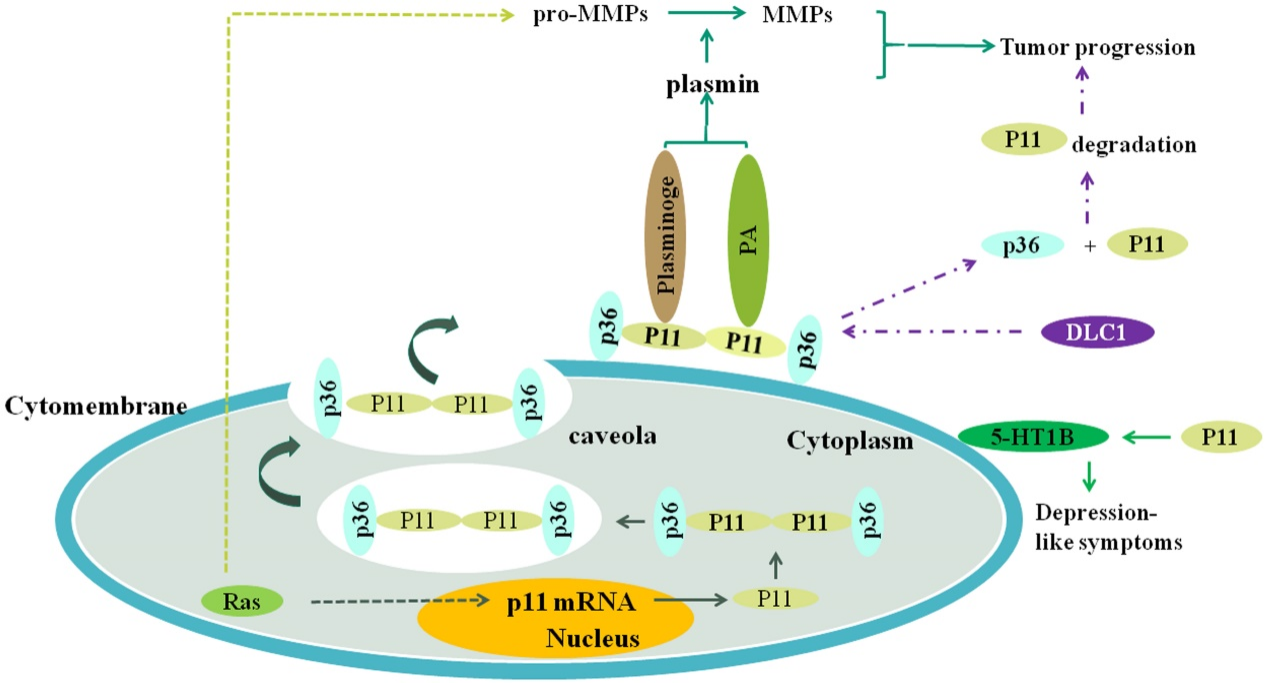
The plasmin is produced when S100A10 (P11) combines to plasminogen and (or) plasminogen activator, and the plasmin can promote the transformation of pro-MMPs to MMPs, such as MMP-2 and MMP-9. Both the plasmin and MMPs collectively promote the tumor progression, including invasion and metastasis. Ras oncogene activates S100A10 expression which activates plasminogen to generate plasmin, and upregulates expression level of plasmin, MMP-2 and MMP-9, which depends on S100A10. DLC1 can competitively combine to S100A10 with annexinA2 (P36), resulting in the separation of P11 and P36 and consequent the ubiquitination degradation of P11, which inhibits tumor progression. S100A10 is associated with depression-like symptoms through affecting the location of 5-HT1B receptor on the cell surface. RAS can increase the transcription of S100A10 through its signaling pathway, resulting in increased expression of S100A10 and consequent generation of plasmin.

The S100 protein family 1, known as glia-specific protein family, belongs to the calcium-binding proteins. It is a super family that consists of at least 20 different members 2, 3, which form the largest subgroup of EF-hand proteins, identified initially by Kretsinger in humans 4. S100 proteins consist of 5 subtypes: S100A, S100B, S100G, S100P, and S100Z. The majority of S100 genes are tightly clustered at chromosome 1q21. Molecular weights of the S100 family of proteins vary within the range of 9 to 13 kDa 5. According to previous studies, S100 proteins are expressed only in vertebrates and similar S100-like sequences may be nonexistent in non-vertebrate eukaryotes 6. S100A10, also known as p11 7-10, is one of the important members of the S100 family, and its regulatory processes *in vivo* are complicated (Figure.1). The most observable physiological and pathological function of S100A10 is the regulation of plasminogen activation and plasmin generation 11, 12, as plasmin production mediated by S100A10 reaches as much as 50% 5. Interestingly, plasmin is involved in fibrinolysis, which depends on the Annexin A2 (p36) or the Annexin A2-S100A10 complex. In the Annexin A2-S100A10 complex, also referred to as AⅡt 13, the S100A10 component is important for plasminogen activation and plasmin production because the production rate of plasmin regulated by Annexin A2-S100A10 complex or S100A10 monomer is more compared to Annexin A2 monomer 14 (Figure.1). Annexin A2 subunit is indispensable for S100A10 stability, although the catalytic rate is low 13. Interestingly, S100A10 plays a diplex-like role in plasmin production. On the one hand, S100A10 protects the newly generated plasmin by preventing α2-antiplasmin-induced plasmin inactivation^14^. On the other hand, S100A10 provokes plasmin autopepsia leading to the destruction of plasmin and generation of plasmin fragments, angiostatins, that are biologically active 5. The S100A10, as a plasminogen receptor, has been shown to participate in extensive regulations in multiple physiological and pathological processes. S100A10 also maintains the normal blood circulation and promotes angiogenesis and metastasis in carcinoma 13.

The plasminogen receptors mostly possess carboxyl-terminal lysine residues 15 that form the binding site for plasminogen and tissue plasminogen activator (tPA). The members of the S100 protein family with carboxyl-terminal lysine residues include S100A4, S100A5, S100A10, S100A13, S100P, and S100Z. However, all of them except S100A10 do not have function as plasminogen receptors. S100A10 is a typical plasminogen receptor, and each monomer of S100A10 can interact with plasminogen. Binding of S100A10 to plasminogen directly activates the transformation of plasminogen to plasmin, thereby inducing proteolysis in the cellular peripheral region. However, the abnormal stimulation of plasmin highly correlates with cancer invasion, metastasis, as well as progression. Apart from S100A10, S100A4 has the ability to bind plasminogen 16. However, whether other amino acid residues participate in the binding of S100A10 to plasminogen or tPA, and whether S100A5, S100A13, S100P, and S100Z proteins activate plasminogen is not yet known 5.

S100A10 plays an important role in a variety of tumor and non-tumor diseases. Here, this review systematically summarizes the structure and functions of S100A10 and its significant role in the modulation of malignant tumor and non-tumor diseases. These underlying molecular events may provide some strategies to target S100A10 expression experimentally.

## The structure, function and complex formation of S100A10

### The structure of S100 proteins family

Many evidences have shown that S100 proteins belong to EF-hand Ca^2+^-binding proteins 3, 5, 17. The S100 family proteins typically comprise two EF-hand motifs: C-terminal canonical EF-hand motif and N-terminal S100-specific EF-hand motif 18. Canonical EF-hand motifs contain 12 amino acid residues, which bind to Ca^2+^ at acidic side-chains. However, the S100-specific EF-hand motifs have 14 rather than 12 residues in which Ca^2+^-binding occurs by one carboxylate side group of glutamic acid 19, 20. Apart from the EF domains, several S100 proteins possess long and flexible carboxyl-terminal extensions, contributing to the specificity of the S100 proteins 1. Moreover, most of S100 proteins could form symmetric noncovalent homodimers, a unique characteristic of the EF-hand family of proteins 5, 21, 22. Interestingly, heterodimers can also be formed between certain S100 proteins in some infrequent instances, like the S100A8/S100A9 heterocomplex 2, 3, 23. Notably, S100G is the only monomer in the S100 protein family 5. When binding to Ca^2+^, all the S100 proteins, except S100A13, undergo a conformational change, exposing many hydrophobic residues of helix I, helix IV, and the hinge region, consequently forming interaction sites for ligands 24.

### S100 proteins family

The S100 proteins are not secreted through the classical Golgi pathway due to the lack of a leader sequence 2. The S100 proteins are implicated in at least 68 interplays with non-S100 targets, possibly benefiting from different conformational changes after binding to Ca^2+^ 25. Functions of S100 proteins have been extensively investigated; their function modes can be intracellular, extracellular, or a combination of both 2, 3. Intracellular roles of all S100 proteins, except S100A15, have been reported. Similarly, extracellular functions of the majority of S100 proteins other than S100A3, S100A16, S100G, and S100Z have been reported 2. Intracellular regulation of multifunctional S100 proteins includes calcium homeostasis, transcriptional factors, cell growth, cell cycle, and phosphorylation. In contrast, extracellular S100 proteins function in a cytokine-like manner via interaction with cell surface receptors, such as the receptor for advanced glycation end-products (RAGE) 26, 27 and the Toll-like receptors (TLRs) 28. In addition, diseases related to the aberrant expression of S100 proteins involve central nervous system disease, heart disease, inflammatory disorders, and tumor progression 1. In human cancers, S100 proteins participate in multiple processes involved in the progression of tumors, such as cancer cell differentiation, proliferation, metastasis, and maintenance of tumor microenvironment.

### Structure of S100A10

**Figure 2:**
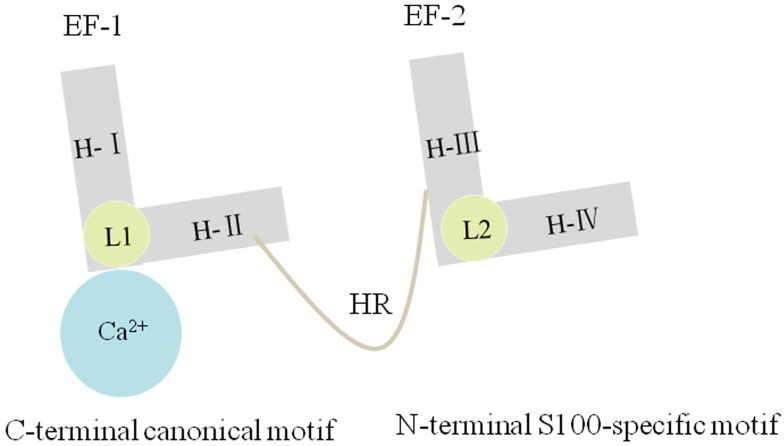
S100A10 has four α-helixes named H-Ι, H-II, H-III and H-IV. The EF-1 domain is composed of H-Ι, H-Ⅱ and L1, and the EF-2 domain is composed of H-III, H-IV and L2. The EF-1 and EF-2 are linked by a hinge region. Three deletions in L1 of S100A10 can render EF-1 domain incapable of binding Ca^2+^.

Each S100A10 monomer consists of four α-helical domains named H-I, H-II, H-III, and H-IV. One EF-hand region of S100A10 is composed of H-I, H-II, and their Ca^2+^-binding loop (L1), which is named as EF-1 domain and is specific for S100 proteins. By comparison, another EF-hand region is made up of H-III, H-IV, and a second loop (L2), which is named as EF-2 domain and is a canonical EF-hand motif. The EF-1 domain and EF-2 domain are connected either by a flexible linker or a hinge region. Three deletions in L1 of S100A10 can render EF-1 domain incapable of binding Ca^2+^ 5, 13 (Figure.2). S100A10 undergoes a mutation, forming a persistently active conformation, similar to the Ca^2+^-on state of other S100 protein members 29, 30.

### Functions of S100A10

As mentioned above, S100 proteins showcase a wide range of interplays with their target molecules, and S100A10 is one such protein 7. S100A10 has been implicated in the recruitment of cationic and anionic channels, covering Na^2+^, K^2+^, Ca^2+^, and Cl^-^ channels 31-33. Reduced sodium current induced by S100A10 was reported to correlate with nociception 34. As a plasminogen receptor, S100A10 positively regulates plasminogen binding and plasmin generation, and its depletion results in a significantly lower fibrinolysis rate as well as an elevated accumulation of fibrin. Furthermore, S100A10 exerts a positive regulatory effect on angiogenesis *in vivo* since its depletion contributes to defective vascularization 35. S100A10 was also shown to suppress the proapoptotic activity induced by Bcl-2-associated death (BAD) promoter 36 and to regulate virus release via complex formation with other proteins 37. Also, S100A10 controls the recruitment of macrophages through activating pro-MMP-9, followed by plasmin-dependent invasion 38 (Figure.1). Monocytes display chemotaxis in response to plasmin, a process dependent on S100A10 39. In contrast, S100A10 can also be regulated by various other factors. For instance, interferon-γ (IFN-γ), glucocorticoid stimulation, neurotrophins 40, and epidermal growth factor (EGF), and Annexin A2 (the characteristic ligand for S100A10) has been reported to coadjust with S100A10. S100A10 mediates the interaction of Annexin A2 with phospholipid membrane by reducing the phosphorylation of Annexin A2 41. Moreover, S100A10 affects the Annexin A2 expression possibly in a tissue-specific way in that the levels of Annexin A2 display a dramatic decline in lung, liver, spleen, and kidney due to S100A10 absence. Notably, the Annexin A2 expression is not affected by S100A10 in the intestine. In turn, Annexin A2 is conducive to the transport and anchoring of S100A10 to the cell surface 12 and imparts stability to the S100A10 homodimer by protecting S100A10 from rapid ubiquitin-mediated degradation. S100A10 is unable to exist in the absence of Annexin A2 and disappears promptly when the cellular Annexin A2 depletes.

### Annexin A2 and S100A10 complex

The Annexin A2-S100A10 complex is composed of two Annexin A2 molecules and S100A10 homodimer. The exact location of the two proteins in the complex has not been determined. It was reported that Annexin A2 and S100A10 were located on the outside and center of the heterotetramer, respectively 42. However, others proposed that S100A10 resided on the outer portion of the complex, thereby facilitating its interplay with other cytoplasmic proteins or receptors 43. The region of amphipathic α-helix located in Annexin A2 amino-terminus is responsible for binding to S100A10 44. The post-translational modification of Annexin A2 amino-terminal domain imparts Annexin A2 acetylation site, serine and tyrosine phosphorylation site, and glutathionylation site. The acetylation of Ser-1 is required for regulating the binding of Annexin A2 to S100A10 45. During this process, the planar Annexin A2 was transformed to a slightly curved disc with a concave and convex side, and the S100A10 binding occurs at the concave side, facing the cytosol 46. The connection sites are housed in H-Ⅰ, H-Ⅳ and the hinge region of S100A10, respectively. There are a total of 19 connection points between S100A10 and Annexin A2 and their specific binding sequences are formed by X-O-O-X-X-O-O-XO, where X refers to hydrophobic residues and O is any other residue 30.

Fundamental studies have identified that the S100A10-ligand complex formation failed due to the replacement of Annexin A2 from the complex Annexin A2-S100A10 and absence of Annexin A2. Therefore, the ligand can function normally only when the ligand interacts with both subunits of Annexin A2-S100A10 complex, and when it has no impact on the binding of S100A10 to Annexin A2. Other S100A10 ligands competing for this binding site with Annexin A2 can result in the subsequent variation of its stability. For instance, deleted in liver cancer-1 (DLC1) is a relatively new ligand for S100A10 and a mediator, negatively regulating cell growth in non-small cell lung cancer (NSCLC) through competitive binding of S100A10 with Annexin A2, resulting in rapid ubiquitin-mediated degradation of S100A10 47. Under physiological conditions, the S100A10 expression is maintained at a steady-state level through the antagonism of DLC1 and Annexin A2 on S100A10 stability. However, in the oncogenic transformation, the levels of DLC1 reduce with the consequent aggrandizement of Annexin A2, and thus, the enhancement of Annexin A2-S100A10 complex becomes uncontrollable. Increase in the Annexin A2-S100A10 complex augments the stability of S100A10 on the cell surface, which leads to the increased generation of plasmin 48.

## The expression S100A10 associates with tumor development

### S100A10 mediates tumor progression through macrophage activities

Conventionally, macrophages are classified into classically- and alternatively-activated macrophages referred to as M1 and M2 subtypes, respectively, which are likely based on distinct expression of their surface markers and functional programs in response to stimuli 49. Tumor-associated macrophages (TAMs) with pro-tumorigenic properties are an emerging subset of macrophages and are closely associated with tumor development and progression. TAMs are involved in the promotion of tumor progression, like mammary cancer and pancreatic carcinoma from multiple profiles containing angiogenesis, migration and invasion, epithelial-mesenchymal transition (EMT), intravasation and extravasation of tumor cells, immunologic suppression, chemoresistance, and immunosuppression 50, and TAM-secreted IL-10 limits the expression of IL-12, which is one of the anti-tumor cytokines 51. S100A10 has been shown to be present on the surface of macrophages 52 and plays important roles in tumor progression induced by TAMs. Macrophages produce plasmin relying on their surface plasminogen receptors, such as S100A10 and histone H2B 53. Plasmin and plasmin-dependent activation of MMP-9 synergistically facilitate the degradation of the extracellular matrix, and thereby, enhance the migration and invasion of macrophages. As expected, this process is severely depressed when S100A10 is absent on the macrophage surface.

### S100A10 cooperates with Ras to regulate tumor development

Ras oncogene 54 is the transgenic gene cloned from two strains of sarcomas, including Harvey and Kirsten in the acute transformational retrovirus experiments. The Ras oncogene participates in the regulation of cell growth and differentiation and the initiation and development of a variety of tumors. Three characteristic genes are known to associate with human carcinomas: H-ras, K-ras, and N-ras located in chromosome 11, 12, and 1, respectively. S100A10 can interact with the Ras oncogene, contributing to the development, invasion and metastasis of cancer. In terms of the mechanisms of tumor invasiveness, S100A10, as a plasminogen receptor, might play a cooperative role with Ras. Some of the plasminogen activators, such as urokinase plasminogen receptor (uPA), and potent S100A10-dependent proteases including plasmin, MMP-2, and MMP-9 can be up-regulated by Ras. Furthermore, plasmin is suggested to simultaneously stimulate the pro-MMP- 1, 3, 7, 9, 10, and 13 that result in the generation of their corresponding MMPs. These proteases promote the invasiveness of tumor cells by degrading and passing through extracellular matrix as well as the basement membrane 55, 56. The expression levels of the members of the plasminogen receptors, such as S100A10 14, S100A4, cytokeratin 8 57, and enolase-1 58 could be influenced by Ras directly. The expression of S100A10 in the presence of Ras is much higher than that without Ras transformation and its target protein Annexin A2 also aggrandizes dramatically. Notably, not all gene sequences of Ras directly regulate the S100A10 expression, and the effector domain mutation responsible for the interaction between S100A10 and Ras may be located in H-Ras. S100A10 is genetically activated by Ras through Ras/RalGDS signaling pathway, which illustrates the importance of Ras in the regulation of the S100A10 gene. Intracellular RAS can increase the transcription and expression of S100A10. The S100A10 and annexin A2 form the S100A10-annexin A2 heterotetramer complex, which is transported to the cell surface via caveola to promote the production of plasmin which plays a key role in Ras-dependent cell invasiveness 55. Congruously, the tumor cells with Ras transformation display a substantial reduction or a loss in the generation of plasmin in the absence of S100A10. The invasiveness of tumor cells sharply decreases in the absence or depletion of S100A10 induced by the reduction of Ras 55.

## The role of S100A10 in different tumors

### S100A10 regulates the initiation of acute promyelocytic leukemia

Leukemia is a kind of malignant disease originating from the hematopoietic stem cells. The cells of leukemia accumulate in the bone marrow and other hematopoietic tissues, which attribute to several relevant mechanisms, including uncontrollable proliferation, failure of benign differentiation, and resistance to apoptosis. The concomitant consequence may be the invasion of leukemia cells to other un-hematopoietic tissues and organs. Therefore, the well-balanced hematopoietic functions have been disrupted by the exceptional proliferation of leukemia cells.

In the acute promyelocytic leukemia (APL) 59, a representative subtype of leukemia, fusion happens through t(15;17) translocation between the retinoic acid receptor α (RARα) gene and the promyelocytic leukemia (PML) gene. Thus, the production of PML-RARα fusion protein is encoded by PML-RARα fusion gene 59. The PML-RARα fusion protein acts as a barrier for the differentiation of promyelocytes into mature and normal promyelocytes. Relatively serious clinical complications of APL contain the disseminated intravascular coagulation (DIC), the pathological process of fibrinous solvation, and the proteolysis induced by proteases. The fibrinolysis process depends on the participation of fibrinolytic system; the fundamental components comprise plasminogen, plasminogen receptor, and plasmin. Without the exception of APL cells, plasminogen receptors are responsible for plasminogen activation and plasmin generation. At the outset, it was considered that Annexin A2 subunit of the Annexin A2-S100A10 complex contributes to the pathological fibrinolysis in APL. Such a result may be stemming from the inhibition of anti-Annexin A2 antibodies to the production of plasmin on APL cells surface, which presents the expression of PML-RARα fusion gene. Therefore, the important roles of S100A10 in APL may be neglected. There also has been confusion whether S100A10 or Annexin A2 plays a role in the incremental generation of plasmin and the succedent excessive fibrinolysis connected with diversified diseases. It has recently been confirmed that S100A10 has a predominant effect on the development of APL. Regarding fibrinolysis and bleeding, the S100A10 has a closer relation to PML-RARα fusion protein, and all-trans retinoic acid (ATRA) compared to Annexin A2. The PML-RARα fusion protein increases the expression of S100A10 on NB4 cells surface. The NB4 cells 60, one of the cell lines of APL, have t(15;17)-translocation necessary for the formation of the PML-RARα fusion gene and constitutively express the PML-RARα fusion protein. In addition, it can also be induced to terminally differentiate into neutrophils with the help of ATRA 60. The direct effects of enhanced S100A10 levels include increased plasminogen activation and excessive fibrinolysis. Correspondingly, the depletion or absence of S100A10 in the NB4 cells results in a dramatic decline in plasminogen activation as well as a much lower migration rate through the fibrin barrier 5. All-trans retinoic acid, one of the metabolites of vitamin A, extensively effects the physiological regulation and pharmacological activity. It could negatively modulate tumors in different manners: first, differentiation and apoptosis of tumor cells can be induced by ARTA; second, ARTA is confirmed to aggrandize the sensibility of tumor cells to chemotherapeutics; third, ARTA facilitates the proliferation of immune cells to reinforce the lethal effect of immune cells on tumor cells. The most significant roles of ARTA for APL may be its induction of terminal differentiation of the leukemic promyelocytes to the mature granular leukocytes, which makes it possible to induce the clinical symptoms of APL to entire remittance. The mechanism of S100A10 interaction with ATRA is not clear. There is a down-regulation or a loss of S100A10, which benefits from the rapid decrease of PML-RARα fusion protein after the management of ATRA 48. Consequently, the activation of plasminogen and plasmin activities suffers a dramatic decline. For APL, down-regulation of both PML-RARα fusion protein and S100A10 are likely responsible for the alleviation of symptoms, such as immoderate fibrinolysis and hemorrhagic complications after the treatment of ATRA.

Collectively, S100A10 protein and PML-RARα fusion gene positively regulate the development and progression in APL, and ATRA is counterproductive to inhibit the progression and alleviate the symptoms. Notably, the subunit of Annexin A2 in the complex with S100A10 plays a less important role than S100A10 in the regulation of APL, although the levels of Annexin A2 increase during the development and progression of APL 61. However, whether Annexin A2 is mainly used to protect S100A10 from ubiquitin-mediated degradation or directly works in the regulation of the PML-RARα fusion protein is yet to be established.

### S100A10 and lung cancer

Lung cancer has high morbidity and mortality rates. Thus, it imposes a threat to health and life. The rate of occurrence of non-small cell lung cancer (NSCLC), a representative subtype of lung cancer, is as high as 80-85%.

S100A10 is well-known for its roles as a plasminogen receptor. In most cases, S100A10 positively regulates the tumor progression through plasmin production and fibrin barrier degradation. Some reports have shown that the overexpression of S100A10 induces a significant increase in lung cancer metastatic sites 8. Although the binding site of S100A10, for its partners is located in the center of the carboxyl terminal sequences, the effects of S100A10 on tumors are diverse, or may be absolutely opposite according to the similarity with the target. The initiation gene of the Rho GTPase-activating protein (RhoGAP), DLC1, is a reversely novel binding target of S100A10. It may be a tumor suppressor gene that negatively regulates the invasion and metastasis of NSCLC by decreasing the expression of S100A10. The S100A10 inhibits the invasion and tumorigenicity of lung cancer cells by its binding to DLC1 in a dose-dependent way 47. Moreover, the reduction of DLC1 induced by gene mutation or genomic transformation directly result in a significant decrease in the production Rho GTPases, a crucial regulator in human oncogenesis 62, 63. Interaction of S100A10 with DLC1 is fairly important for human NSCLC, especially, for the suppression of cell growth, proliferation, invasion, and metastasis as well as in the facilitation of apoptosis. These inhibiting effects are dependent on the formation of the S100A10-DLC1 complex, and a hydrophobic amino acids domain within the sequences aa340-630 of DLC1 is responsible for binding to S100A10. Furthermore, a 7-amino acid peptide STFNNVV, located in aa348-354 within the sequences aa340-630 may be binding to S100A10 more specifically because DLC1 fails to bind to S100A10 without this peptide 47. Interestingly, the hydrophobic domain of DLC1 is highly analogical to the binding region of Annexin A2 to S100A10 44. Therefore, DLC1 is able to interact with S100A10 through competing with the carboxyl-terminal sequence with Annexin A2. Many studies have suggested that a single S100A10 monomer is quite labile and Annexin A2 can function as a protector by preventing S100A10 protein from ubiquitin-dependent degradation because Annexin A2 confers a ubiquitin ligase acting site to S100A10 64. However, the S100A10-protective ubiquitin ligase acting site disappears when S100A10 interacts with DLC1. In addition, interaction of DLC1 with S100A10 also affects the activation of plasminogen via occupying two lysine residues necessary for S100A10 binding to plasminogen. Hence, the rapid decline in the S100A10 protein may be largely attributed to the increasing expression of DLC1. Lower fibrinolysis or production rate of plasmin mainly benefits from the reduced S100A10, which works as a vital plasminogen receptor 8, 65. Legitimately, DLC1 partly inhibits invasion and metastasis of NSCLC through its negative regulation for S100A10.

Together, the decline of S100A10 expression induced by its binding to DLC1 and its consequent ubiquitin-dependent degradation decreases or loses the activation of plasminogen to plasmin. Moreover, the carboxyl-terminal lysine residues of S100A10, used for binding to plasminogen is occupied and allosterically altered by DLC1; therefore, the activation of plasminogen to plasmin is also reduced. Both of the above processes partially inhibit the invasiveness and metastasis in NSCLC.

### S100A10 in other solid tumors

It is established that S100A10 plays an indirect, but important role in the fibrinolytic system, which belongs to a protective physiological response of the organism through its immediate stimulation to plasminogen. S100A10 participates in both invasion and metastasis of tumor cells by activating proteases, such as plasmin, MMP-2, MMP-9, as well as their receptors. The plasmin stimulated by S100A10 can also directly promote the activation of MMPs. Therefore, S100A10 is a pivotal regulatory factor for tumor progression, especially for the invasiveness and metastasis of tumor cells. Breast cancer, for instance, is positively regulated by S100A10 and its target protein Annexin A2 in the migration of its tumor cells 66. For pancreatic ductal adenocarcinoma whose five years survival rate is especially low in pancreatic cancers, it is most likely that the S100A10 gets highly up-regulated, and rather than Annexin A2, plays an important role in its increased invasiveness 67. Besides, all of the components of Annexin A2-S100A10 complex (S100A10 and Annexin A2) are up-regulated in renal cell carcinoma. However, the specific regulatory mechanisms of S100A10 in renal cell carcinoma are not very clear 68. In addition, S100A10 gene in the Akt/NF-κB signaling pathway potentially involves in the initiation of hepatocellular carcinoma 69. It is surprising that the expression levels of S100A10 and its target protein Annexin A2 in prostate cancer is significantly reduced or absent 70. However, there are also some researchers who hold the opposite view that S100A10 and Annexin A2 are positively correlated with the malignancy of prostate cancer 71. Generally, there is not a specific conclusion that how S100A10 influences prostate cancer. Several studies have proved that the expression of S100A10 was closely related to the sensitivity of colorectal cancer to chemotherapy drug oxaliplatin (L-OHP). The colorectal cancer cells with a high expression of S100A10 show a low sensitivity to L-OHP 72, 73. It is different from the chemosensitivity of colorectal cancer cells to 5-Fluoracil (5-FU) of which chemosensitivity cannot be reflected by the expression of S100A10 protein 74. More importantly, it reflects that S100A10 may be a potential as well as relatively specific biomarker resistant to L-OHP in colorectal tumor patients. Several documents indicate that this may be due to the impact of S100A10 on the apoptotic activity of Bcl-xL/Bcl-2 associated death promoter (BAD) for tumor cells. This also reflects the promotion of S100A10 to the progression of colorectal cancer from the side.

## The role of S100A10 in non-tumor diseases

### Potential mediatory roles of S100A10 in the depression disorder

Depression, whose occurrence and development are influenced by both genetic factors and the environment, is one of the most complex mental disorders. Patients suffering from depression display many obvious changes, such as lasting and bad emotion, cognitive impairment and abnormal behavioral phenotype. A plethora of related neurotransmitters and proteins are involved in the regulation of depression. For instance, the 5-hydroxytryptamine (5-HT), a teleorganic amine, exerts multifarious effects on central nervous system, partially in that it has various subtypes including 5-HT1, 5-HT2, and so on. Furthermore, each subtype of 5-HT might interact with specific receptors 75 and the 5-HT1B receptor is an important regulatory factor that is capable of participating in the mediation of some mental disorders, like depression and anxiety. Several reports have shown that the depression disorder is regulated by the 5-HT1B receptor. Recently, it was documented that S100A10 is potentially able to be involved in the regulation of depression 76 through its distribution in the brain that is relative to the depression-like behavioral phenotype 76 or interacting with 5-HT1B receptor 77. S100A10 distributes widely in the vertebrate body containing the brain in which the expression of S100A10 is subsistent in some neurons and nonneuronal cells as well as is discrepant within disparate regions. It is possible that the distribution of S100A10 in the brain is correlated with specific behavioral symptoms of depression. For instance, the lateral hypothalamus cells with positive expression of S100A10 might modulate abnormal food intake by secreting orexin 78. The loss of S100A10 expressed in ChAT neurons of nucleus accumbens is likely to induce the anhedonia or despair. Besides, S100A10 in the paraventricular nucleus of the hypothalamus plays a potential and integral role in the regulation of the stress response, which might benefit from several hormones, such as vasopressin and corticotropin releasing hormone (CRH) 79. In addition, S100A10 located in the glutamatergic neurons of the forebrain may be related to the susceptibility of the depression disorder and the depletion of S100A10 within this region is more accessible to depression under relatively slight stressor 80. However, it is unexpected that the number of cells that express S100A10 in each region appears to not perform a key role in behavior. Furthermore, it has been described that the permeability as well as the structure of the hematoencephalic barrier composed of endothelial cells and astrocytes are changed in depression 81. Nevertheless, whether S100A10 exerts an essential role and how it works in this process are not yet known.

Apart from that, the interaction between S100A10 and 5-HT1B receptor is also closely relevant to the depression-like states 77. The release of 5-HT is inhibited by the 5-HT1B receptor, which acts as an autoreceptor. Meanwhile, the reduced release of glutamic acid in the integrant neurons of the cerebral cortex might be influenced by a combination between 5-HT and 5-HT1B receptor. Interestingly, S100A10 has been proven to increase the colocalization with 5-HT1B receptor on the cell surface. In the absence of S100A10, the metabolites of 5-HT are expectedly elevated, which may be induced by the decreased localization of 5-HT1B receptor at the cell surface, and several depression-like phenotypes also display increased thigmotaxis and immobility as well as decreased horizontal activities. Moreover, the levels of S100A10 in the brain are up-regulated after management with antidepressants. Taken together, both distribution and the number of cells expressing S100A10 in the brain play a mediatory role in depression disorder. However, the mechanisms through which S100A10 interacts with the 5-HT1B receptor and their attributions to depression remain a mystery, which requires further investigations.

### S100A10 in inflammation-related actions

Macrophages that derive from monocytes and differ from TAMs have a central effect on the pathological inflammatory responses, such as atherosclerosis, arthritis, and restenosis 82, 83. Recruitment and migration through ECM of peripheral macrophages to the inflammatory site requires plasmin and other proteinases 84, 85.

Of note is the generation of plasmin that originates from the conversion of plasminogen, an inactive zymogen, by activating the activator, tPA and uPA 86, 87. Furthermore, plasmin possesses the capacity of direct activation for MMP-1, MMP-3, and MMP-13 as well as indirect stimulation of MMP-2 and MMP-9, thereby augmenting degradation of and migration rate through extracellular matrix 88. S100A10 and its target protein Annexin A2 have been identified on the surface of macrophages and both of them play an important role in response to inflammatory stimuli. It is known that S100A10, a plasminogen receptor, promotes the generation of plasmin and its carboxyl-terminal lysine residues are crucial binding sites for plasminogen kringle domains. The treatment of macrophages with carboxypeptidase B, a digestive enzyme cleaving the carboxyl terminal lysine, shows a decreased generation of plasmin and a dramatically reduced invasion through the matrigel 89. Generation of plasmin by macrophages from mice with knockdown of S100A10 decreased up to approximately 45% 38. Namely, knockdown of S100A10 in macrophages compromises their capacity of plasminogen binding; thereby, declaring that S100A10 is not the only one plasminogen binding protein that depends on lysine and is involved in the degradation of extracellular matrix and basement membrane. The identification of the concrete molecules participating in the migration and invasion of macrophages remains elusive. In addition, depletion of S100A10 induces a diminished recruitment of macrophages, impairing the ability of movement through the barrier of the basement membrane of the macrophages; thus, indicating that S100A10 plays an important role in macrophage migration. For MMPs activated by plasmin, MMP-9 has been verified that plays a vital role when macrophages migrate through matrigel 90 and the deficiency of S100A10 leads to a declining activation of proteinase MMP-9.

Other than S100A10, Annexin A2, α-enolase, and histone 2B on the macrophages surface have been suggested to work as plasminogen receptors and play an essential role 53, 91, 92 in the recruitment of the macrophages to the inflammatory site in that the S100A10-absent macrophages; they have limited capability of plasminogen binding and plasmin generation under treatment with carboxypeptidase B. In terms of Annexin A2, considered as the most prominent target protein of S100A10, its expression severely decreases on S100A10-absent macrophages surface. Annexin A2 has been controversially suggested to be conducive for the activation of plasminogen and generation of plasmin at the macrophages surface 93. Previous investigations have shown that the structurally intact Annexin A2 proteins are incapable of binding to plasminogen 94. Only the processed form of Annexin A2, which results from the carboxyl-terminal cleavage of the intact Annexin A2 by proteinases and exposes a new C-terminal lysine, could bind plasminogen and modulate the plasmin generation. The proteinases involved in this process are not yet explicit, but plasmin has been ruled out 95. However, only intact Annexin A2 was detected at the cell surface of the macrophages in inflammatory responses; thus, elaborating that these Annexin A2 proteins do not directly participate in the plasminogen binding. In contrast, another conversion of Annexin A2 induced by plasmin 84 exposes an amino-terminal peptide with an ability to interact with transmembrane receptors, which leads to a series of intracellular molecule signaling cascades and a pro-inflammatory response 84 by activating several cytokines including TNF-α, IL-1, and IL-6. Moreover, transcriptional factors NF-γB has been induced to translocate to nucleus and several mitogen-activated protein kinases (MAPK) were phosphorylated by the amino-terminal peptide of Annexin A2 in macrophages, which promotes the release of inflammatory cytokines and chemokines as well as confers macrophages increasing bacteria-phagotrophic efficiency 96. Furthermore, the JAK-STAT signaling pathway was also activated by stimulating typeⅠ cytokine receptors caused by this N-terminal peptide, which induces an elevated expression of monocytes and macrophage chemoattractant protein 1 (MCP-1) 97. The release of these inflammatory cytokines also induces the recruitment of pro-inflammatory cells like macrophages and monocytes, which potentially play an essential role in the inflammatory diseases.

## Conclusions

**Figure 3:**
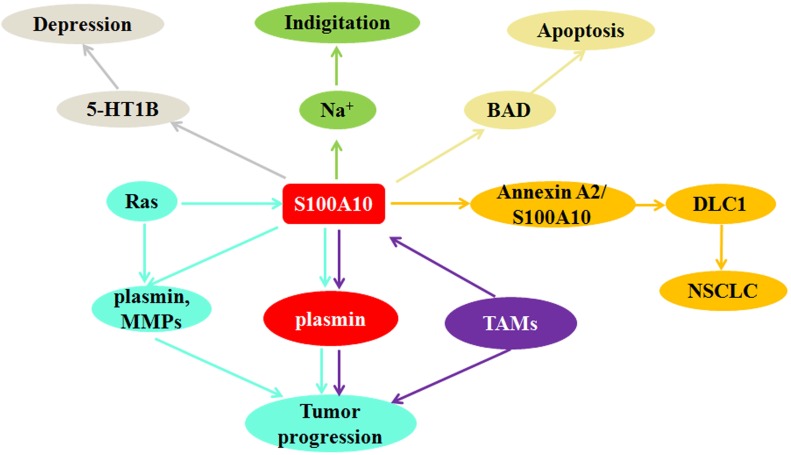
S100A10 can regulate indigitation through down-regulating Na^+^ current; S100A10 mediates depression-like state by affecting the localization of 5-HT1B receptor on cell surface; S100A10 supresses apoptosis through inhibiting Bcl-2-associated death promoter (BAD) activation; DLC1 competitively binds S100A10 with annexin A2 to inhibit invasion and metastasis of NSCLC; Ras and TAMs promote tumor progression via regulating S100A10 expression.

S100A10 was known to be involved in the regulation of various malignant tumors and non-tumor diseases. In this study, we reviewed S100A10 modulates tumor progression through macrophage activities and Ras signaling pathway, including the promoting role in the initiation and development of acute promyelocytic leukemia and lung cancer. Furthermore, the potential mediatory roles of S100A10 in non-tumor diseases were also discussed (Figure. 3). S100A10 expression in brain could improve the depression-like behaviors, and S100A10 regulated several inflammatory responses at the surface of macrophages to enhance the ability of movement through impairing the barrier of the basement membrane, contributing to macrophages recruitment in inflammation.
